# Dominance of HIV-1 Subtype CRF01_AE in Sexually Acquired Cases Leads to a New Epidemic in Yunnan Province of China

**DOI:** 10.1371/journal.pmed.0030443

**Published:** 2006-11-14

**Authors:** Yong Zhang, Lin Lu, Lei Ba, Li Liu, Li Yang, Manhong Jia, Haibo Wang, Qing Fang, Yuhua Shi, Wenyun Yan, Guangcai Chang, Linqi Zhang, David D Ho, Zhiwei Chen

**Affiliations:** 1 Yunnan Center for Disease Control and Prevention, Kunming, Yunnan, People's Republic of China; 2 Aaron Diamond AIDS Research Center, Rockefeller University, New York, New York, United States of America; 3 State Key Laboratory of Virology, Modern Virology Research Center and AIDS Center, Wuhan University, Wuhan, Hubei, People's Republic of China; Johns Hopkins University, United States of America

## Abstract

**Background:**

Dating back to the first epidemic among injection drug users in 1989, the Yunnan province has had the highest number of human immunodeficiency virus type 1 (HIV-1) infections in China. However, the molecular epidemiology of HIV-1 in Yunnan has not been fully characterized.

**Methods and Findings:**

Using immunoassays, we identified 103,015 accumulated cases of HIV-1 infections in Yunnan between 1989 and 2004. We studied 321 patients representing Yunnan's 16 prefectures from four risk groups, 11 ethnic populations, and ten occupations. We identified three major circulating subtypes: C/CRF07_BC/CRF08_BC (53%), CRF01_AE (40.5%), and B (6.5%) by analyzing the sequence of *p17,* which is part of the *gag* gene. For patients with known risk factors, 90.9% of injection drug users had C/CRF07_BC/CRF08_BC viruses, whereas 85.4% of CRF01_AE infections were acquired through sexual transmission. No distinct segregation of CRF01_AE viruses was found among the Dai ethnic group. Geographically, C/CRF07_BC/CRF08_BC was found throughout the province, while CRF01_AE was largely confined to the prefectures bordering Myanmar. Furthermore, C/CRF07_BC/CRF08_BC viruses were found to consist of a group of viruses, including C, CRF08_BC, CRF07_BC, and new BC recombinants, based on the characterization of their reverse transcriptase genes.

**Conclusions:**

This is the first report of a province-wide HIV-1 molecular epidemiological study in Yunnan. While C/CRF07_BC/CRF08_BC and CRF01_AE are codominant, the discovery of many sexually transmitted CRF01_AE cases is new and suggests that this subtype may lead to a new epidemic in the general Chinese population. We discuss implications of our results for understanding the evolution of the HIV-1 pandemic and for vaccine development.

## Abstract 2

This is a molecular epidemiology study of circulating HIV strains and subtypes in Yunnan province, which has China's largest number of HIV-infected individuals.

## Editors' Summary

**Background.:**

The first human immunodeficiency virus (HIV) cases in China were seen in 1989 in Yunnan, a region of south-western China. This area borders Myanmar, Laos, and Vietnam, and is a major entry point for illegal drugs into China. The initial HIV outbreak in this area was in injecting drug users, but HIV is beginning to affect other groups of people in the Yunnan province and is becoming more common across China. There is still not much known about the different types of HIV virus in China and which parts of the population are most likely to be infected. This knowledge is important because it can help people to understand how the epidemic started and how it is likely to spread in the future, and because it helps direct efforts for HIV education and prevention. It is also necessary for the future design of appropriate HIV vaccines.

**Why Was This Study Done?:**

The Yunnan province has the highest rate of HIV-infected individuals in China. It is an important entry point of new HIV virus types into China, and some of the HIV types found in patients in other parts of China appear to have spread from Yunnan. A group of researchers from the United States and China wanted to look at the different types of HIV virus that were infecting people in the Yunnan province and to work out how these types had evolved over the course of the HIV epidemic in China. They focused on human immunodeficiency virus type 1 (HIV-1), the most common form of HIV virus worldwide and also the most infectious. There are at least nine distinct subtypes of HIV-1, and the virus continues to evolve and to form new subtypes.

**What Did the Researchers Do and Find?:**

They collected blood samples from 321 HIV-infected individuals who represented a broad cross-section of the population of Yunnan (people from all geographic parts of the province, 11 ethnic populations, different occupations, etc.) and analyzed the genetic information of the viruses found in these blood samples. Because HIV evolves very rapidly, the genetic information differs between different virus subtypes, and the researchers could therefore tell which subtypes were infecting which subsets of the population. The researchers identified three distinct subtypes of HIV-1: “B” (in about 6.5% of the samples), a group of “C” variants (C/CRF07_BC/CRF08_BC in 53% of the samples), and CRF01_AE (in 40.5%). The CRF01_AE subtype had not previously been reported at such high levels in the Chinese population, and people who were thought to have been infected with HIV through sexual contact (as opposed to contaminated needles) were more likely to be infected with that particular subtype.

**What Do These Findings Mean?:**

The results show a dynamic and evolving pattern of HIV types in the Yunnan province, segregating among different parts of the population. Sexual transmission appears to be on the rise, suggesting that the epidemic could spread rapidly from high-risk groups such as drug users to the general population. HIV/acquired immunodeficiency syndrome (AIDS) education and prevention efforts in the general population are therefore urgently needed. It is also likely that some of the developments of the HIV epidemic in the Yunnan province will be similar in other parts of China as the various subtypes spread. The results of the study also have implications for future HIV vaccine development. Given the range of subtypes, it will be necessary either to develop vaccines that can protect against all the circulating subtypes, or to have a cocktail of several vaccines that each protects against some of them.

**Additional Information.:**

Please access these Web sites via the online version of this summary at http://dx.doi.org/10.1371/journal.pmed.0030443.

- Information from AVERT, an international AIDS charity on HIV subtypes and HIV in China
- The UNAIDS on AIDS in Asia
- The China AIDS Network—a charity devoted to AIDS research in China

## Introduction

**Figure 1:**
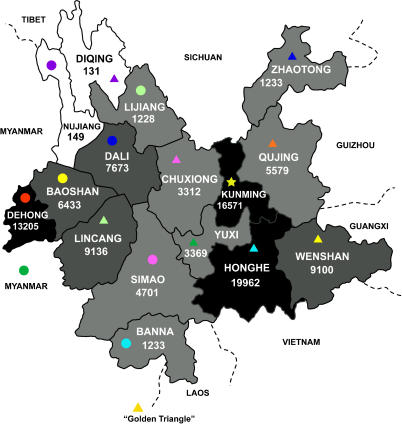
Map of the Yunnan Province of the People's Republic of China and Distribution of Reported Cases of HIV-1 Infection in 2004 The color-coded symbols represent the geographic origins of HIV-1 strains as depicted in Figures 2, 3, and 6.

Located in south-western China, Yunnan has a total of 16 prefectures including 129 counties and cities with an estimated total population of 42.9 million. Out of 16 prefectures, eight prefectures (26 counties) border Myanmar (1,997 km of border), Laos (710 km of border), and Vietnam (1,353 km of border) (Figure 1). Owing to its geographic proximity to the “Golden Triangle,” where Myanmar (Burma), Thailand, and Laos converge and much of the world's heroin is manufactured, Yunnan serves as an important entry point for drug smuggling to China and beyond [1,2].

The initial human immunodeficiency virus type 1 (HIV-1) outbreak in China was detected among injection drug users (IDUs) in Ruili City in the Dehong prefecture of the Yunnan province in 1989 [3–5]. One hundred and forty-six infected individuals were initially identified, setting off an alarm that a true human immunodeficiency virus (HIV)/acquired immunodeficiency syndrome (AIDS) epidemic in China had begun. Since these individuals were mainly from the Dai minority group (80%), there was a tendency of HIV-1 segregation among this ethnic population during the early stages of the epidemic. Heroin use and needle sharing were the major risk factors, giving HIV-1 an easy means of transmission. This led to the HIV-1 prevalence rate reaching approximately 89% among some groups of IDUs within Yunnan, with a provincial average of about 25% [6]. As a result, Yunnan rapidly became the most severely affected province within China [6,7]. In recent years, it has become of increasing concern that HIV-1 has begun to spread from the IDU into other high-risk and general populations. The HIV-1 prevalence rate among commercial sex workers, pregnant women, and gay men has been reported to have increased over the past few years [6]. A previous report described that the majority of infections (14/15) via sexual contact in Yunnan were due to B and C/CRF07_BC/CRF08_BC subtypes, with the exception of one CRF01_AE infection [8]. Therefore, the genetic characterization of HIV-1 among other at-risk populations and their relationship with those in the IDU population remained to be investigated.

Over the past few years, there have been several reports on the characterization of HIV-1 genotypes in Yunnan, largely confined to the IDU population. Multiple HIV-1 subtypes have been identified, including B, B′, C, E (now called CRF01_AE), CRF07_BC, CRF08_BC, and unique recombinant form (URF) [8–17]. Furthermore, some of these subtypes of HIV-1 have spread through drug trafficking, not just to the neighboring provinces of Guangxi and Sichuan but also to the north-western province of Xinjiang [18–21]. We recently reported that subtype B′ viruses found among paid blood donors in and around Central China were closely related to viruses previously identified in Yunnan [7,22]. This forms the basis for the hypothesis that the viruses that disseminated widely into many other regions and populations of China originated in Yunnan. Therefore, we undertook a comprehensive study to define HIV-1 genotypes that are common in the Yunnan province. Here, we report the findings from what we believe to be the first large-scale, province-wide molecular epidemiological study of HIV-1 infection in Yunnan.

## Methods

### The Study Participants and Specimens

A total of 863 HIV-1–positive serum samples were collected between 2002 and 2004 from individuals at the local HIV/AIDS sentinel surveillance sites in Yunnan. The study was conducted in a cross-sectional and anonymous way according to guidelines set by the local ethical review committee at the Yunnan Center for Disease Control and by the Rockefeller University Institutional Review Board. HIV-1 infection status was determined by an enzyme immunoassay and confirmed by a Western blot assay [6]. As there is no capacity for separating and storing human peripheral blood mononuclear cell samples at the local laboratories, only serum was used to obtain HIV-1 RNA for subsequent analysis. The modes of transmission for these individuals mainly include intravenous drug use, sexual contact, blood transfusion, and mother-to-child transmission (Table 1). For statistical analysis, we performed the chi-square test of association or the Fisher's exact probability test.

**Table 1:**
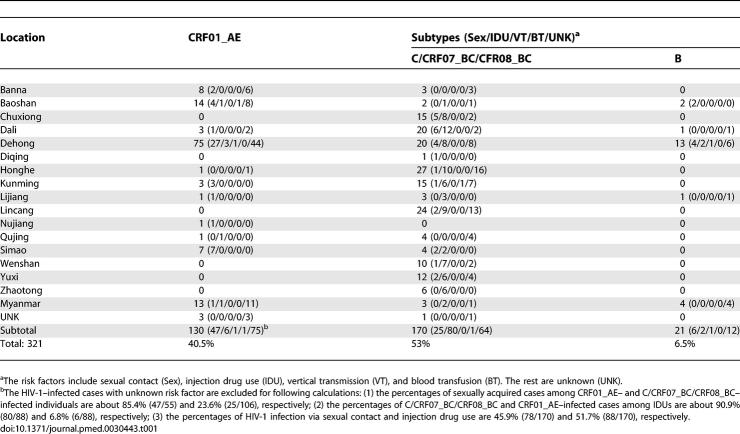
The Geographic Distribution of HIV-1 Subtypes in Yunnan, China

### Amplification of HIV-1 Gene Fragments by Reverse Transcription Polymerase Chain Reaction

Viral RNA was extracted from the patients' serum using the QIAamp Viral RNA Mini kit (Qiagen, Valencia, California, United States). The viral RNA was then subjected to reverse transcription polymerase chain reaction (RT-PCR). For the *gag* gene, the primer for reverse transcription (RT) reaction was 5′-TCTAATACTGTATCATCTGCTCCTG (antisense), which is HIV-1–specific and was successfully used for all major subtypes found in China [7,22]. A 485-bp *gag* fragment covering the *p17* gene was amplified by polymerase chain reaction (PCR) with nested primers using the Expand High Fidelity PCR System (Roche, Indianapolis, Indiana, United States). The *p17* gene was selected because it had previously been used for HIV-1 phylogenetic analysis and appeared less prone to recombination [7,22,23]. PCR primers for the *p17* gene are 5′-TCTCTCGACGCAGGACTCGGCTTG (sense) and 5′-TCTAATACTGTATCATCTGCTCCTG (antisense) for the first-round reaction, and 5′-TTTGACTAGCGGAGGCTAGAAG (sense) and 5′-GCTCCTTCTGATAATGCTGAAAACATGGG (antisense) for the second-round reaction.

To study the viral recombination, we also amplified RT gene fragments from a subset of randomly selected samples. In this case, random hexamer was used for the RT reaction. PCR primers used for a 413-bp RT fragment are 5′-TTAAAGCCAGGAATGGATGG-3′ (sense) and 5′-GCCTCTGTTAATTGTTTTACATCATTAGTGTG-3′ (antisense) for the first-round reaction, and 5′-GTTAAACAATGGCCATTGACAG-3′ (sense) and 5′-GCCTCTGTTAATTGTTTTACATCATTAGTGTG-3′ (antisense) for the second-round reaction. Moreover, PCR primers used for a 1,049-bp RT fragment are 5′-TTAAAGCCAGGAATGGATGG-3′ (sense) and 5′-TGGAATATTGCTGGTG-ATCCTTTCC-3′ (antisense) for the first-round reaction, and 5′-GTTAAACAATGGCCATTGACAG-3′ (sense) and 5′-TGCTGGTGATCCTTTCCATCC-3′ (antisense) for the second-round reaction. The amplification cycles are 95 °C for 2 min followed by 35 cycles of 95 °C for 15 s, 55 °C for 45 s, and 72 °C for 1.5 min plus the last extension of 72 °C for 8 min. Amplified PCR products were purified using a QIAquick PCR purification kit (Qiagen) and subjected to DNA sequencing directly by an automated ABI 377 DNA sequencer (Applied Biosystems, Foster City, California, United States).

### Phylogenetic Analysis of HIV-1 Sequences

To eliminate potential contamination, all of the sequences obtained were first subjected to an HIV-1 Blast search to compare with related reference sequences in the HIV Databases, funded by the Division of AIDS of the National Institute of Allergy and Infectious Diseases (NIAID), a part of the National Institutes of Health (NIH) (http://hiv-web.lanl.gov/content/index). Nucleotide sequences were aligned with the references using the Clustalx 1.81 program, and further adjusted manually [24,25]. The genetic distance of HIV-1 sequences analyzed was calculated using Kimura's two-parameter model [26]. Phylogenetic trees were generated using the neighbor-joining method, implemented in the Clustalx 1.81 program. The branch significance was analyzed by bootstrap with 1,000 replicates. The trees were printed using the TreeView program [27]. The bootscan analysis was performed using SimPlot 3.5 software kindly provided by S. Ray [28].

## Results

### HIV-1/AIDS in Yunnan by 2004

Dating back to the first HIV-1 epidemic among IDUs in 1989, the virus has spread throughout the province. All 16 prefectures in Yunnan have reported cases of HIV-1 infection (Figure 1). These reported cases tested serum-positive for HIV-1 by an immunoassay at the local sentinel surveillance sites and were further confirmed by a Western blot assay at the Yunnan Center for Disease Control. By the end of 2004, we had found an accumulated total of 103,015 reported cases in the province and 1,223 reported cases of AIDS, 744 of whom had died. These numbers made Yunnan the most severely affected province in China, accounting for 30% of nationally reported HIV-1 infections, 26% of AIDS cases, and 28% of deaths due to HIV/AIDS. The geographic distribution and the breakdown of these reported cases are depicted in Figure 1. The most severely affected prefectures (shaded in black) include Dehong, Kunming, and Honghe, where more than 10,000 infected cases had been identified by the end of 2004.

### Characteristics of Study Participants for the Molecular Epidemiological Study

To understand the characteristics of HIV-1 molecular epidemiology in Yunnan, we conducted a comprehensive study on patient samples collected across the entire province from 2002 to 2004. For this study, serum samples were subjected to RT-PCR to recover HIV-1 genes. Using this method, we were able to obtain 321 HIV-1 *gag p17* genes from the sera of 863 patients from the 16 prefectures. The relatively low amplification rate (321/863; 37.2%) is probably related to low viral load in some cases, but is more likely due to RNA degradation owing to poor serum storage, transport conditions, or sequence variations at the primer binding sites. More samples from Dehong have been tested because it was the starting point of the HIV-1 epidemic in China as well as being a severely affected prefecture (Figure 1). The geographic distributions of these 321 positive samples are summarized in Table 1. These positive samples were subjected to further analysis. Based on the patients' records, only 170 study participants had clear documentation for their risk factors including injection drug use (88/170; 51.7%), sexual contact (78/170; 45.9%), mother-to-child transmission (2/170; 1.2%), and blood transfusion (2/170; 1.2%) (Table 1). The rest were unknown. Most (85.9%) of our study participants were between 18 and 45 y of age with an average age of between 21 and 35, suggesting that the majority of the labor force and sexually active people are more vulnerable to infection. Among the remaining participants, 1.2% were younger than 18 y and 7.5% were older than 45 y. The gender ratio of our study participants was 2.3:1 (222:95, male:female), which is slightly lower than that of 2003 (2.61:1) based on the total number of infections in Yunnan [6].

Yunnan's ethnic diversity is unrivalled in China, with 25 different minority groups representing one third of its 42.9 million people. Besides the Han ethnic group that accounted for the majority of our study participants (189/321; 58.9%), HIV-1 was detected in ten other minority populations including Dai (17.8%), Jing-Po (3.4%), Hui (2.8%), Bai (1.9%), Yi (1.9%), Wa (0.6%), Bu-Yi (0.6%), Yao (0.3%), Na-Xi (0.3%), and E-Chang (0.3%). The rest (10.9%) were unknown. The occupations of our study participants included farmers (46.7%), unemployed (29.6%), self-employed (6.9%), factory workers (6.2%), female sex workers (1.9%), civil servants (2.2%), truck drivers (1.2%), students (0.9%), migrant workers (0.9%), children (1.2%), army soldiers (0.6%), and unknown (1.6%). Based on the characteristics described above, the participants studied not only represented the high-risk groups, but also included a significant proportion of the general Chinese population.

### Identification of Three Major Subtypes of HIV-1 in Yunnan

**Figure 2:**
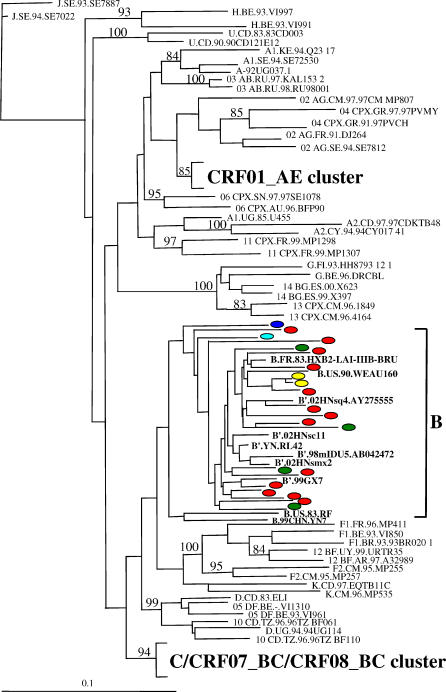
Phylogenetic Neighbor-Joining Tree for HIV-1 *p17* Sequences Obtained from All 16 Prefectures of Yunnan Individual sequences are color coded, with the colors corresponding to those of original geographic sites on the map of Yunnan (Figure 1). The horizontal branch was drawn in accordance with their relative genetic distances. The vertical lines are present purely for clarity of the tree presentation. The bootstrap values of 1,000 replicates are labeled on the major branches. The reference sequences for classifying HIV-1 genotypes were included and were originally obtained from the NIH/NIAID–funded HIV Databases.

**Figure 3:**
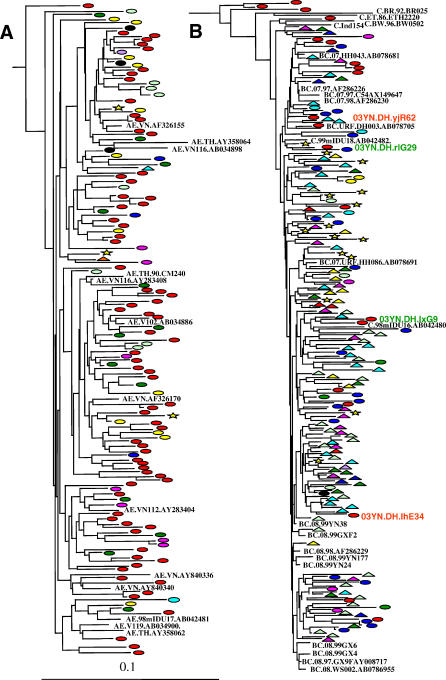
Continuation of the Phylogenetic Neighbor-Joining Tree Shown in Figure 2 Two clusters represent the two major circulating HIV-1 subtypes in Yunnan: CRF01_AE (A) and C/CRF07_BC/CRF08_BC (B).

The RT-PCR amplicons were directly sequenced and the data were subsequently used for phylogenetic analysis to study the genetic relationship among the viruses in Yunnan, and with those in other regions of China and around the world. We chose the HIV-1 *gag p17* gene for this study mainly because it has been commonly used for HIV-1 genotyping as previously described [7,22]. Because *gag p17* does not have sequence-length polymorphism, this approach does not involve gene cloning and therefore is practical for local use [7,22]. The reference sequences were obtained from the NIH/NIAID–funded HIV Databases covering the major HIV-1 subtypes and circulating recombinant forms (CRFs) (Figure 2). Moreover, for the purpose of comparison, we included some reference sequences which were previously characterized in China and in countries surrounding Yunnan, including Vietnam, Thailand, and Myanmar [29]. We also studied 20 infected individuals who were Myanmar nationals but were found living in Yunnan. Based on phylogenetic analysis using a neighbor-joining method, we found that there are three major HIV-1 subtypes circulating in Yunnan: C/CRF07_BC/CRF08_BC (170/321; 53%), CRF01_AE (130/321; 40.5%), and B (21/321; 6.5%) (Table 1; Figures 2 and 3). It is evident that CRF01_AE and C/CRF07_BC/CRF08_BC are the two major subtypes likely to be codominating the current HIV-1 epidemic in Yunnan. The prevalence of C/CRF07_BC/CRF08_BC is not surprising given previous reports [9–14]. However, the high prevalence of CRF01_AE is a new finding.

We also estimated the evolutionary divergence for sequences obtained within and between each geographic location by analyzing the genetic distances using the Kimura two-parameter model, which allows for different rates of transition and transversion. Interestingly, despite a smaller number of infections identified, the mean genetic distance of subtype B viruses (8.4%) is larger than those for subtypes CRF01_AE (6.9%) and C/CRF07_BC/CRF08_BC (5.3%). This finding suggests that subtype B viruses probably hit Yunnan earlier than other subtypes and continued to evolve over time. It is also possible that multiple diverse subtype B viruses were introduced into Yunnan. Interestingly, there are three Dehong C/CRF07_BC/CRF08_BC viruses that branch out on the top of the phylogenic tree (Figure 3B). These divergent viruses are probably derived from some new B/C recombinants or from separate clade C viral introductions. Furthermore, as the mean genetic distance of subtype CRF01_AE viruses is larger than that of C/CRF_07/CRF08_BC, we speculate that this CRF01_AE epidemic may not be a recent development in Yunnan. It may also reflect the possibility that many diverse CRF01_AE viruses were introduced into Yunnan across the Myanmar border simultaneously. This notion was evident when several equally divergent CRF01_AE viruses were found among Myanmar people now living in Yunnan (Figure 3A).

### Distinct Distribution of Two Major Subtypes of HIV-1 among Different Risk Groups

Although our study participants engaged in multiple forms of risky behavior, injection drug use and sexual contact (96% combined) were the two major factors for HIV-1 infection (Table 1). Interestingly, HIV-1 strains from a given prefecture did not appear to cluster together in the phylogenetic tree. For example, viruses of subtype CRF01_AE from the Dehong prefecture were found on many branches of the tree instead of clustering tightly together (Figure 3A). Similar observations hold true for viruses derived from other prefectures or from subtype C/CRF07_BC/CRF08_BC (Figure 3A and 3B). These results also indicate that the introduction of these viruses into each prefecture was not likely to be the result of a single event. However, the overall distribution of the two major HIV-1 subtypes, C/CRF07_BC/CRF08_BC and CRF01_AE, tend to differ by geographic location.

**Figure 4:**
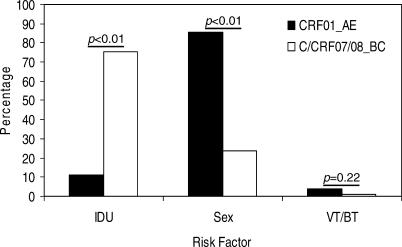
The Distinct Distribution of Two Major HIV-1 Subtypes among Different Risk Groups in Yunnan The risk factors include sexual contact (Sex), injection drug use (IDU), vertical transmission (VT), and blood transfusion (BT). The *p-*values are based on analysis using the chi-square test of association or Fisher's exact probability test.

Although C/CRF07_BC/CRF08_BC was found throughout the entire province, this viral genotype appears to largely dominate the eastern region of Yunnan, which is close to the Guangxi province, where a CRF08_BC epidemic was previously reported [21]. This region includes the three most heavily affected prefectures: Honghe, Kunming, and Wenshan (Figure 1). In contrast, CRF01_AE viruses appear to be geographically confined to the western region including Banna, Dehong, and Baoshan, all bordering Myanmar (Figures 1 and 3A). Importantly, the distribution of these two major subtypes is closely associated with transmission route. We found that among those who had CRF01_AE infections, 85.4% (47/55) were infected through sexual contact (Table 1). In contrast, among those who had C/CRF07_BC/CRF08_BC infections, 76.2% (80/105) were infected through injection drug use (Table 1). Strikingly, 90.9% (80/88) of IDUs had C/CRF07_BC/CRF08_BC infections, whereas only 6.8% (6/88) of IDUs had CRF01_AE infections (Table 1). Statistically, the percentage of C/CRF07_BC/CRF08_BC infections was significantly higher than that of CRF01_AE infections among IDUs (*p* < 0.01, Figure 4). Conversely, the percentage of CRF01_AE infections was significantly higher than that of C/CRF07_BC/CRF08_BC infections among sexually transmitted cases (*p* < 0.01, Figure 4).

These findings suggest that the risk factors have played a significant role in determining the spread of these two distinct and dominant viral subtypes in Yunnan. In view of the fact that CRF01_AE viruses have become dominant in the sexually transmitted population, this finding should alert experts to the possibility of a new wave of HIV-1 epidemic largely targeting the general population. This situation is serious because a large percentage of HIV-1 infections were found among farmers (150/321) instead of sex workers. For CRF01_AE infections alone, 53.2% (25/47) of the sexually transmitted cases were farmers, indicating the severe dissemination of this subtype HIV-1 among the general Chinese population. It should be noted that farmers still represented more than 57.01% of China's 1.3 billion people in 2005.

To understand whether there is an ethnic group segregation of HIV-1 CRF01_AE subtype among the Dai people, we investigated the distribution of three viral subtypes among different ethnic populations. We found that the majority of these sexually transmitted CRF01_AE cases were among the Han (25/47) and Dai (15/47) populations. However, the proportional difference of sexually transmitted cases in these two ethnic groups did not reach statistical significance (*p* = 0.26). Therefore, these findings suggest that the spread of CRF01_AE viruses is confined neither to the Han nor to the Dai ethnic groups. Among sexually transmitted Dai individuals, because the proportion of CRF01_AE cases (15/47) is significantly higher than that of C/CRF07_BC/CRF08_BC (2/25) (*p* < 0.05), we cannot exclude the possibility that the infection of the Dai people might have contributed to the sexual transmission of CRF01_AE viruses. Further studies will be needed to investigate this issue.

**Figure 5:**
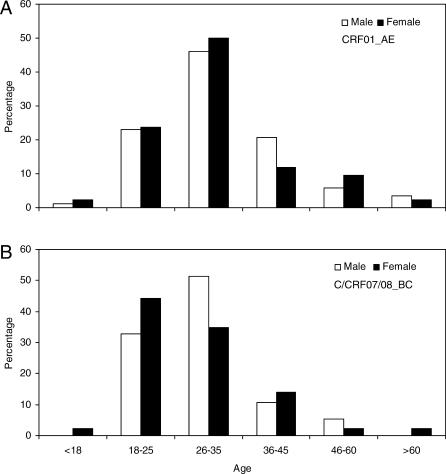
Two Major HIV-1 Subtypes among Different Age and Gender Groups in Yunnan The upper panel represents subtype CRF01_AE (A), whereas the lower panel is for C/CRF07_BC/CRF08_BC (B). The *y*-axis indicates the percentage of HIV-1 infection in each age and gender group. There were significantly more females at age 18–25 infected by C/CRF07_BC/CRF08_BC than by CRF01_AE viruses (*p* < 0.05).

To determine whether the percentage of CRF01_AE infections is significantly higher in the younger generation, we compared the distribution of infected individuals based on their age and gender groups (Figure 5). We did not find a significant difference between CRF01_AE– and C/CRF07_BC/CRF08_BC–infected male populations (*p* > 0.05). We did, however, find that there were significantly more females at age 18–25 infected by C/CRF07_BC/CRF08_BC than by CRF01_AE viruses (*p* < 0.05). We could not determine which risk factor had contributed to this observation because the majority of these study participants did not provide sufficient information.

In this study, we also analyzed 20 HIV-1–infected people from Myanmar who actually lived in the Dehong prefecture near the border area. We found that their viruses, particularly subtype CRF01_AE strains, are genetically indistinguishable from those found in Dehong (Table 1; Figures 1–3). In fact, the viruses previously identified in Myanmar, Vietnam, and Thailand are also closely related to the viruses characterized here. Considering the close geographic proximity, we believe that these viruses that group closely in the phylogenetic trees must share common ancestors (Figures 2 and 3). As the CRF01_AE virus was previously found in Myanmar and Vietnam, it is very likely that these viruses were transmitted from there and then disseminated in Yunnan .

### Coexistence of HIV-1 Subtype C, CRF07_BC, CRF08_BC, and New BC Recombinant Strains in Yunnan

**Figure 6:**
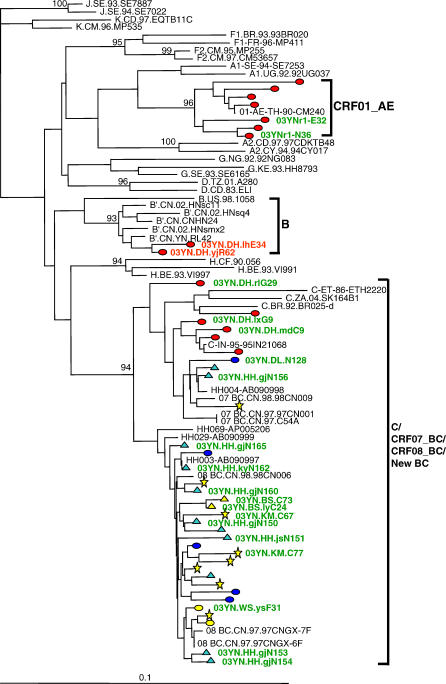
Phylogenetic Neighbor-Joining Tree for HIV-1 RT Fragment Sequences Obtained from 43 Individuals Individual sequences are color coded, with the colors corresponding to those of original geographic sites on the map of Yunnan (Figure 1). The horizontal branch was drawn in accordance with their relative genetic distances. The vertical lines are present purely for clarity of the tree presentation. The bootstrap values based on 1,000 replicates are labeled on the major branches. The reference sequences for classifying HIV-1 genotypes were included and were originally obtained from the NIH/NIAID–funded HIV Databases.

Since the sequence of *gag p17* does not differentiate between the infections of subtype C, CRF07_BC, and CRF08_BC, we further amplified a 413-bp fragment of HIV-1 RT genes from 43 randomly selected study participants. These 43 participants were from the prefectures Dehong, Dali, Honghe, Kunming, Baoshan, and Wenshan where C/CRF07_BC/CRF08_BC infections are dominant among IDUs (Figures 1 and 3). We chose the RT gene because it contains genetic makeup to distinguish previously defined C/CRF_07/CRF_08_BC viruses. We included seven CRF01_AE–infected individuals from Dehong as experimental controls including four sexually transmitted cases, one IDU, and two with unknown risk factors. Based on the phylogenetic analysis, these seven Dehong CRF01_AE viruses did not show inter-subtype recombination. Their RT genes remain clustered with the CRF01_AE virus in the phylogenetic tree (Figure 6). Surprisingly, 36 C/CRF07_BC/CRF08_BC viruses, which were previously defined by the *gag p17* sequences, displayed complicated genetic features in their RT genes.

In Dehong, RT genes derived from study participants represent a more highly divergent group of viruses than those found in other prefectures (Figure 6). Among the eight Dehong individuals studied (four IDUs, two sexually transmitted cases, and two with unknown risk factors), two viruses, 03YN.DH.lhE34 and 03YN.DH.yiR62, were found grouped tightly with B′ viruses in the RT tree representing likely new forms of BC recombination (*p17* C/RT B′). The other six viruses are relatively closely related to subtype C strains. In fact, four of the six viruses clustered tightly with the reference India subtype C virus, indicating the lack of BC recombination in the sequences studied. This finding supports the hypothesis that Indian and Chinese subtype C viruses share a common origin. Interestingly, two sexually transmitted cases were infected with pure C, although their viral full-genome sequences remain to be studied. The apparent lack of pure CRF08_BC or CRF07_BC viruses in Dehong probably reflects the fact that viruses there have undergone further recombination, which is made evident by a bootscanning analysis (see below). Our findings support the notion that Dehong served as a site for multiple diverse viruses to enter Yunnan and as a unique geographic location to foster new viral recombination among IDUs. In contrast, unlike in Dehong, in other prefectures a relatively homogeneous group of CRF08_BC and CRF07_BC viruses are the dominant forms of circulating recombinant viruses among IDUs. In particular, there were more CRF08_BC viruses found among IDUs with only one sexually acquired case. To some extent, this finding probably indicates a new trend of CRF08_BC epidemic among IDUs in Yunnan.

**Figure 7:**
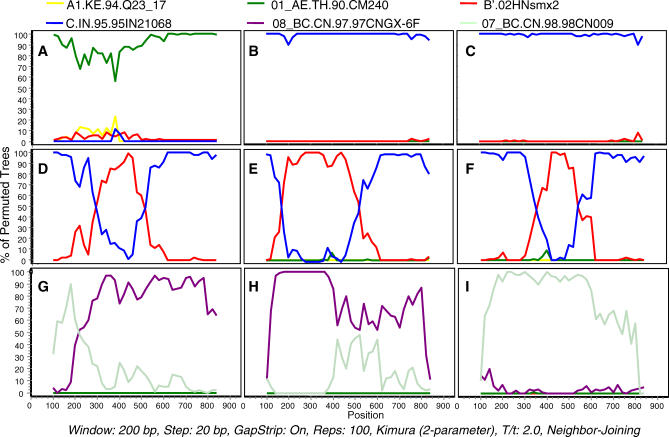
Bootscanning Analysis of HIV-1 RT Sequences of Six Study Participants Participants included one CRF01_AE virus 03YN.DH.rlE32 (A), two pure subtype C viruses 03YN.DH.mdC9 (B) and 03YN.DH.lxG9 (C), one new BC recombinant virus 03YN.DH.rlG29 (D and G), one CRF08_BC virus 03YN.HH.gjN165 (E and H), and one CRF07_BC virus 03YN.HH.gjN156 (F and I). The reference sequences obtained from the NIH/NIAID–funded HIV Databases are color-coded and shown on the top. The conditions used for this analysis are shown at the bottom. The boostrap values are based on 100 replicates using the neighbor-joining method.

To further study the issue of viral recombination, we were able to recover a 1,049-bp fragment of HIV-1 RT genes from 17 study participants. These participants are color-coded, with their names in green in the phylogenetic tree (Figure 6). Their viral sequences were subjected to a bootscanning analysis [28]. Consistent with the phylogenetic analysis, Dehong CRF01_AE and pure subtype C viruses did not have inter-subtype recombination (Figure 7). Moreover, all CRF08_BC or CRF07_BC viruses had typical recombination features that are similar to those previously reported (Figure 7). For the viruses found in Dehong, we found another virus that is likely a new BC recombinant virus in addition to the two new BC recombinant viruses 03YN.DH.lhE34 and 03YN.DH.yiR62. This virus, 03YN.DH.rlG29, has a somewhat long branch in the cluster of C, CRF08_BC, or CRF07_BC viruses in the phylogenetic tree (Figure 6). Moreover, as shown in the bootscanning analysis (Figure 7), this virus has a distinct break point before the position 300 at the 5′ side of its RT gene. By doing bootscanning analysis against CRF07_BC and CRF08_BC, we found that this virus is likely to be a URF with its RT gene derived from the hybridization between CRF07_BC and CRF08_BC viruses (Figure 7). It should be noted that all three new BC recombinant viruses were found among IDUs in Dehong.

## Discussion

In this study, we conducted what we believe to be the first large-scale prefecture-by-prefecture molecular epidemiological study of HIV-1 infection in Yunnan, the province most severely affected by HIV-1/AIDS in China. Based on the genetic characterization of HIV-1 *gag p17* sequences of 321 samples collected between 2002 and 2004, we mapped the distribution of HIV-1 subtypes throughout this area. We found that HIV-1 subtypes C/CRF07_BC/CRF08_BC and CRF01_AE predominate in Yunnan, accounting for 53% and 40.5% of HIV-1 infections, respectively. Because 85.4% of CRF01_AE infections were acquired through sexual contact, while 90.9% of IDUs had C/CRF07_BC/CRF08_BC viruses (Table 1), the distribution of these two major subtypes is likely to be related to the mode of viral transmission rather than to ethnic segregation. Interestingly, C/CRF07_BC/CRF08_BC viruses were found to consist of a group of viruses, including C, CRF08_BC, CRF07_BC, and new BC recombinants, based on the characterization of their RT genes.

The changing patterns of the dominant HIV-1 subtypes in Yunnan indicate the complex evolving dynamic nature of the epidemic. Dating back to the identification of the first HIV-1 epidemic in the Dehong prefecture in 1989, the average prevalence rate of HIV-1 infection among IDUs increased from about 7% before 1995 to 23% afterwards. [6,30,31]. A massive screening test conducted in 2004 identified 13,486 infected patients alone (unpublished data). This number accounts for a 90% increase compared to that identified in 2003, despite the fact that the true incident rate remains unclear [6]. The rapid expansion of the epidemic has resulted in the identification of reported cases of HIV/AIDS in each of the 16 prefectures (Figure 1). At the end of 2004, Yunnan had 103,015 reported cases of HIV-1 infection and 1,223 cases of AIDS; 744 of these patients with AIDS had died. These numbers made Yunnan the most severely affected province, accounting for 30% of nationally reported HIV-1 infections, 26% of AIDS cases, and 28% of related deaths [6].

While the virus spread rapidly throughout the Yunnan province, the viral subtype of the dominant epidemic strains also evolved in an unexpected dynamic way. The following reports were based on the characterization of HIV-1 strains derived from IDUs in the Dehong prefecture. In 1989, the subtype B virus was found to be the leading causative agent for the first HIV-1 epidemic in Yunnan [6,32]. Two years later, subtype B′ strains were reported to have become the major epidemic strain [10,33]. Thereafter, a rising epidemic of Indian subtype C viruses was reported [12]. From our study, it is clear that none of these early strains of HIV-1 plays a major role in the epidemic today. Although the driving force for the viral selection remains unclear, the coexistence of multiple genotypes among the same IDU population has provided an ideal setting for the emergence of recombinant HIV-1 strains.

Intravenous drug use is the major risk factor that drives the evolution of diverse types of viral recombination in Yunnan. Consistent with previous findings, we found that the recombinant forms CRF07_BC/CRF08_BC are likely to be predominant among IDUs throughout Yunnan [13,14,17,34,35]. There is, however, a clear trend that additional recombinant viral variants are continuously emerging in Yunnan. Previous studies reported the findings of multiple URFs likely due to the superinfection of IDUs by CRF07_BC and CRF08_BC viruses in the Honghe prefecture [16]. Recently, a study on five nearly full-length HIV-1 genomes of IDUs at Ruili City in the Dehong prefecture showed that none of those viruses share an identical viral genome [14]. In contrast to the genome structure of CRF07_BC and CRF08_BC viruses, three new Dehong URFs displayed a reverse genomic organization with subtype C segments in the clade B backbone. Each of these three new URFs had a *p17* C/RT B combination in their genome.

Here, we have reported the findings of three additional URFs. Interestingly, two of our URFs had the *p17* C/RT B combination in their genome. Because these two individuals were found outside Ruili City, one in Ying-Jiang and one in Liang-He, our data indicated that this kind of virus had probably been spreading throughout the Dehong prefecture among IDUs. Moreover, our finding of the new URF, 03YN.DH.rlG29, from an IDU in Ruili City has provided additional evidence that new recombinant viral variants are continuously emerging among IDUs in Dehong. Lastly, our finding of several India-like subtype C viruses not only suggested that the India and Chinese C viruses shared a common origin but also provided evidence that they were the actual source of subtype C sequences in the circulating BC recombinant viruses in Yunnan. Taken together, our findings have revealed the complex viral evolution among IDUs in Yunnan. Given the fact that some IDUs are infected with CRF01_AE viruses, it is likely only a matter of time before the new recombinant viruses between AE and BC recombinant viruses are found. Studies on full viral genome sequences are required to further understand the characteristics of viral recombination.

The rising number of HIV-1 CRF01_AE infections is likely to be related to the change of risk groups over the past few years in Yunnan. Based on previous findings and data obtained in this study (Table 1), it is not surprising to note that B and CRF07_BC/CRF08_BC viruses can be sexually transmitted in Yunnan, particularly in prefectures where there is a lack of CRF01_AE infection, because the transmission could be due to the viruses originally harbored by local IDUs [15]. However, our finding of a large number of sexually transmitted CRF01_AE viruses in Yunnan among non-IDUs is new. In particular, the rapid increase of HIV-1 prevalence among non-IDU populations has emerged as an alarming trend [6].

IDU dominance in the late 1980s and throughout the 1990s has now been gradually replaced by those infected through heterosexual contact or other routes [6,36,37]. First, there was an increasing proportion of HIV-1 infections due to sexual transmission—from less than 5 % in 1996 to up to 20% recently [6]. Patients with sexually transmitted disease and female sex workers are the two major components for this outcome [6,38]. Second, the infected population has greatly shifted from ethnic minorities (Dai and Jing-Bo) to the Han majority population [6,39]. The percentage of HIV-1 infections among Dai and Jing-Bo dropped from more than 80% in 1989 to about 50% in 1994. Nowadays, the Han population accounts for up to 80% (58.9% in this study) of the infections [6]. Third, a large proportion of infected farmers had begun to be replaced by unemployed or self-employed personnel, rural-to-urban migrant workers, and factory workers, which accounted for up to 50% (43.9% in this study) of the infected population [6]. This transition coincided with the HIV-1 spread from rural to urban areas. Lastly, HIV-1 prevalence among pregnant women also increased from less than 0.2% in 1995 to 0.38% recently [6]. Altogether, we believe that these changes have accelerated the spread of HIV-1 infection among the non-IDU populations.

Here, we have provided the direct evidence that subtype CRF01_AE has dominated the rising number of HIV-1 infections acquired sexually. As this finding relied on non-IDUs and there was only one documented female sex worker infected with the CRF01_AE virus, we believe that this viral subtype has already invaded Yunnan's general population. It is important to closely monitor Yunnan's non-IDUs at prefectures where the CRF07_BC/CRF08_BC virus is currently dominating. It is also crucial to monitor the whole country's general population in a similar manner. This is a big concern as a separate study recently revealed that subtype CRF01_AE very likely led to a separate epidemic in another region of China, mainly through sexual transmission [40].

Understanding the new trend of molecular epidemiology of HIV-1 infection would be critical for vaccine development in Yunnan. With the increasing number of HIV-1 infections via the route of heterosexual transmission, there is no doubt that the development of a safe, effective, and affordable vaccine will be the ultimate solution for China. For vaccine development, one critical stage is to determine the efficacy of an HIV-1 vaccine candidate in a human population. With a sufficient number of newly infected cases each year, Yunnan may serve as a potential site for vaccine testing. In this context, our data will assist the selection of vaccine testing sites. For the testing of a C/CRF07_BC/CRF08_BC–based vaccine, for example, one may want to conduct a study in the areas with the fewest CRF01_AE–infected individuals if the goal is for the determination of subtype-specific protection. However, if the goal includes the test for broad, cross-subtype protection, the areas with CRF01_AE–infected people would be acceptable. In any case, continuous monitoring of the circulating HIV-1 subtypes would be essential for directing future vaccine trials in Yunnan.

We believe that the control of the sexual transmission of HIV-1 in the world's most populated nations will have a significant impact on the war against the HIV/AIDS pandemic. The present study was carried out in the province most heavily affected by HIV/AIDS in the world's most populated nation. Our study reveals an actively ongoing epidemic caused by the CRF01_AE virus which has not been previously reported. When we review the current situation of HIV/AIDS in China, two major epidemics are not due primarily to sexual transmission. Instead, injection drug use leads to the spread of the CRF07_BC/CRF08_BC virus among IDUs throughout almost the entire country, while paid blood donation gives rise to the dissemination of clade B virus in Central China and surrounding provinces [7,22]. Worst of all, each of these events has resulted in many hundreds of thousands of infections. As HIV-1 is mainly a sexually transmitted virus, we believe that our findings should set off a loud alarm that the spread of the CRF01_AE virus should be controlled. With the rapid expansion of this virus subtype in Yunnan and other regions in China and its surrounding countries largely via sexual activities, we believe that our findings will have implications for the understanding of the new trend of the HIV pandemic, not only in China but also in Asia and the rest of the world.

## Supporting Information

### Accession Numbers

The GenBank (http://www.ncbi.nlm.nih.gov/Genbank) accession numbers for the nucleotide sequences discussed in this paper are DQ915980–DQ916022 and EF061985–EF062305.

## Abbreviations

AIDS: acquired immunodeficiency syndrome
CRF: circulating recombinant form
HIV: human immunodeficiency virus
HIV-1: human immunodeficiency virus type 1
IDU: injection drug user
NIAID: National Institute of Allergy and Infectious Diseases
NIH: National Institutes of Health
PCR: polymerase chain reaction
RT: reverse transcription
RT-PCR: reverse transcription polymerase chain reaction
URF: unique recombinant form
